# Orthopedic interventions and outcomes in patients with metastatic renal cell carcinoma: a systematic review

**DOI:** 10.1177/17588359251385393

**Published:** 2025-10-15

**Authors:** Abdulrahman Al Bochi, Mohamad Tarek Madani, Muhammad El-Kassem, Mohammed Fadel, Adnan Rajeh, Gabriel Boldt, Ricardo Fernandes

**Affiliations:** Schulich School of Medicine and Dentistry, Western University, London, ON, Canada; Schulich School of Medicine and Dentistry, Western University, London, ON, Canada; Schulich School of Medicine and Dentistry, Western University, London, ON, Canada; Schulich School of Medicine and Dentistry, Western University, London, ON, Canada; Verspeeten Family Cancer Centre, Victoria Hospital, London Health Sciences Centre, London, ON, Canada; Division of Medical Oncology, Department of Oncology, Schulich School of Medicine & Dentistry, Western University, London, ON, Canada; Division of Experimental Oncology, Department of Oncology, Schulich School of Medicine & Dentistry, Western University, London, ON, Canada; Department of Oncology, London Health Sciences Centre, Western University, 800 Commissioners Road East, Room A3-940, London, ON N6A 5W9, Canada; Division of Medical Oncology, Department of Oncology, Schulich School of Medicine & Dentistry, Western University, London, ON, Canada

**Keywords:** bone metastasis, orthopedic interventions, palliative therapy, radiotherapy, renal cell carcinoma, targeted therapy

## Abstract

**Background:**

Despite advanced in systemic therapy for renal cell carcinoma (RCC), bone metastasis remains an adverse prognostic factor and major cause of mortality and morbidity. Orthopedic interventions may provide symptom relief, functional recovery, and survival benefit, yet the evidence is fragmented across heterogeneous studies.

**Objectives:**

To systematically review the outcomes of orthopedic surgical interventions in patients with symptomatic bone metastases from RCC.

**Design:**

Systematic literature review conducted in accordance with Preferred Reporting Items for Systematic Reviews and Meta-Analyses guidelines.

**Data sources and methods:**

A comprehensive search of Embase, MEDLINE, and the Cochrane Library was conducted for studies published between January 1, 2014, and January 30, 2024. Eligible studies included randomized controlled trials, prospective observational studies, and retrospective studies reporting on orthopedic interventions for RCC bone metastases. Primary outcomes included overall survival, reoperation rates, complication rates, and quality of life.

**Results:**

Of 2208 studies screened, 15 met the inclusion criteria. All were retrospective series or case series, limiting the strength of the evidence. Six studies focused on appendicular skeleton metastases, five on axial skeleton involvement, and four on both regions. Results were primarily reported narratively, with limited statistical analyses. Orthopedic surgical interventions—particularly when combined with targeted systemic therapy—were associated with longer overall survival. Among surgical approaches, complete metastasectomy was most consistently associated with improved survival compared with intralesional curettage and stabilization-only procedures.

**Conclusion:**

Although available data suggest that orthopedic surgery, particularly complete metastasectomy, may improve overall survival and quality of life in RCC patients with bone metastases, the evidence is limited to retrospective and narrative reports. Some studies also suggest that outcomes may be further enhanced when surgery is integrated with systemic therapy. Given the poor prognosis associated with bone involvement in RCC, prospective randomized studies are urgently needed to define optimal patient selection, standardize management strategies, and integrate surgery with systemic therapy in a multidisciplinary framework.

## Plain language summary

Bone surgery and treatment results in people with kidney cancer that has spread to the bones: a summary of research studies

Why we did this study: Renal cell carcinoma (RCC) is a type of kidney cancer that can spread to the bones, causing pain and reducing quality of life. Given the fact that treatments for RCC have improved, patients are living longer, which means that managing bone-related complications has become more challenging and important. We wanted to find out whether orthopedic surgery helps patients with bone metastases from RCC live longer or feel better compared to other treatments like radiation or medications. What we did: We reviewed and analyzed research studies published between 2014 and 2024 that looked at treatments for RCC patients with bone metastases. We focused on comparing orthopedic surgery, particularly surgeries that remove cancer from the bone to other approaches like radiation therapy, medications, or simpler bone stabilization procedures. We looked at outcomes such as survival, complications, the need for further surgery, and quality of life. What we found: Out of more than 2,000 studies, we included 15 in our review. We found that patients who had surgery to completely remove bone tumors (called complete metastasectomy) tended to live longer than those who had less aggressive procedures or non-surgical treatments. Combining surgery with targeted cancer drugs may offer even more benefit. Radiation therapy may help in some cases; however, the evidence is limited. Most of the studies found were not randomized trials, which means the results are not definitive. What this means: Orthopedic surgery, particularly when combined with other treatment, can help some patients with RCC that have spread to the bones live longer and improve their quality of life. However, more high-quality studies are needed to confirm these benefits and to help doctors decide which patients will benefit the most from surgery.

## Introduction

Renal cell carcinoma (RCC) is the most lethal urologic malignancy, accounting for 2%–3% of all adult malignant tumors.^1^ In 2021, RCC represented 76,080 new cases and over 13,000 deaths in the United States of America (USA).^2^ Approximately 20%–30% of patients present with metastatic disease at diagnosis.^3,4^ The bones (30%–40% of RCC patients) are one of the most common sites for metastasis, along with lungs (50%–60%), liver (30%–40%), and more rarely, brain (5%).^5^

Historically, targeted therapies such as vascular endothelial growth factor (VEGF), tyrosine kinase inhibitors (TKIs), and mammalian target of rapamycin (mTOR) inhibitors improved survival in metastatic RCC from approximately 1–2 years.^6–8^ In recent years, the treatment paradigm has shifted with immune checkpoint inhibitor (ICI)-based combination regimens, including ICI–ICI and ICI–TKI doublets, becoming the standard of care for treatment-naïve mRCC.^9–12^ These combinations have demonstrated survival benefits across risk groups, and subgroup analyses of pivotal randomized controlled trials (RCTs) have included patients with bone metastases. However, outcomes in this subgroup remain poorer than in patients without bone involvement,^13–15^ confirming that bone metastases are an established adverse prognostic factor.

Bone metastases are widely recognized as an unfavorable prognostic site in RCC, associated with worse survival compared to metastases at other sites, even in the modern treatment era.^16^ Bone metastases from RCC are predominantly osteolytic,^17^ occurring most commonly in the long bones (femur and humerus) and axial skeleton.^18,19^ They cause significant morbidity through the occurrence of skeletal-related events (SREs), including pain, pathologic fractures, spinal cord compression, and hypercalcemia.^20^ Bone metastases may present synchronously at initial diagnosis or metachronously during follow-up, and their prognostic implications differ.^21^ Similarly, patients with a single bone lesion often have better outcomes than those with multiple sites,^22^ although both scenarios remain indicative of systemic disease, and local therapy decisions must be made within the context of overall oncologic management.

Therapeutic options for bone metastasis include systemic therapy, radiotherapy, bone-seeking isotopes, bisphosphonates, and Rank-L ligands (i.e., denosumab).^23–27^ Orthopedic interventions are generally palliative, aiming to restore structural stability, alleviate pain, enable early mobilization, and prevent or treat pathological fractures.^28^ While retrospective series have suggested possible survival benefits from complete metastasectomy in selected patients,^29^ these findings are based on small, highly selected cohorts and may reflect confounding from baseline prognostic factors rather than a causal benefit. No prospective trial has demonstrated a survival advantage for surgery in bone metastases from RCC, and available data are predominantly retrospective or case series, often reported narratively with limited statistical analysis.

In North America, the increase in survivorship of patients with RCC indicates that more orthopedic surgical interventions may be warranted in patients with bone metastasis.^30,31^ Investigating the prevalence of individuals could allow us to understand the scope of implications of cancer survivorship. Given the shift to modern ICI-based combination therapy and the lack of high-level evidence for surgical intervention, there is a need to re-evaluate the role of orthopedic surgery in the multidisciplinary management of bone metastases in RCC. We therefore conducted a systematic review of the literature to compare orthopedic surgical interventions with other palliative treatments, including radiotherapy, in patients with symptomatic bone metastases from RCC. Our aim was to assess survival, complications and reoperation rates, and quality-of-life outcomes, while contextualizing findings within the modern systemic therapy era and acknowledging the limitations of the existing evidence base.

## Methods

### Design

This study was designed as a systematic literature review conducted in accordance with the Preferred Reporting Items for Systematic Reviews and Meta-Analyses (PRISMA) guidelines.^32^ The completed PRISMA checklist is provided as Supplemental File 1. The review aimed to evaluate outcomes of orthopedic interventions in patients with RCC and bone metastases, with a focus on survival, complications, reoperation rates, and quality of life. The protocol was structured using the Population, Intervention, Comparator, Outcomes, Study Design (PICOS) framework to guide eligibility and synthesis.

### Information sources and search strategy

For this review, EMBASE, MEDLINE, and Cochrane Library databases were searched for relevant studies from January 1, 2014 to January 30, 2024, to summarize the most recent available evidence. The search strategy was developed by one author (AA) in consultation with a health information specialist (GB) using the following key terms and their derivatives: “Renal Cell Carcinoma” and “Metastasis” and “Bone” or “Orthopaedics” (see Supplemental content for full search string).

### Selection process and eligibility criteria

This systematic review was designed to address the following research question: “are there differences in clinical outcomes between orthopedic and other treatment interventions in patients with symptomatic bone metastases from RCC?”

We applied the PICOS framework to structure the research question and its corresponding literature search. The population of interest was patients with history of renal cell carcinoma and bone metastasis. The intervention was an orthopedic intervention to manage bone metastasis (BM). The outcomes included: overall survival, incidence of reoperations, surgical complications, and quality of life. In terms of study design, eligible studies included randomized controlled trials, prospective observational studies, and retrospective studies. We excluded case reports, narrative reviews, conference abstracts, and studies not written in English due to resource limitations for translation. All references obtained from the search strategy were uploaded into the Covidence systematic review management software,^33^ an online platform designed to facilitate screening, data extraction, and conflict resolution in systematic reviews, thereby improving transparency and reproducibility in study selection. Two authors (AA, MTM, ME, MF, RF) independently screened titles and abstracts, followed by full-text review, using a two-step screening process. Disagreements were resolved by consensus, with a third author consulted if necessary. Reasons for exclusion at the full text stage were documented in the PRISMA flowchart.^32^

### Data collection and extraction

A standardized data extraction form was developed in consultation with the study team and pilot-tested on two studies. Two authors (AA, MTM, ME, MF) independently extracted data through Covidence.^32^ Conflicts in data extracted between the two authors were resolved by consensus, and a third author arbitrated when consensus was not reached. For each included study, we collected data on study and population characteristics, interventions, outcomes, and key takeaways. Study characteristics included country of origin, study objectives, study methodology. Sample characteristics included size, age, male to female ratio, number of bony metastastic sites, RCC histology subtype, and pattern of metastatic disease (other than bone metastasis). Interventions included type (i.e., surgical method +/− adjuvant therapy), treated bone metastasis site, and use of bisphosphonates or denosumab. Overall survival (OS) was the primary outcome of interest for this review, but others such as reoperation rates, complication rates, and quality of life were extracted when available.

### Synthesis of results

We conducted a narrative summary of the findings, presented alongside a flow diagram and table of study characteristics. To organize the results, studies were grouped according to the anatomic site of treated bone metastasis (axial skeleton; appendicular skeleton; mixed). A meta-analysis was not performed due to the data heterogeneity in patient population, outcomes, and variability in the type, distribution, and reporting of systemic treatment regimens across studies.

## Results

### Selection of included articles

**Figure 1.**
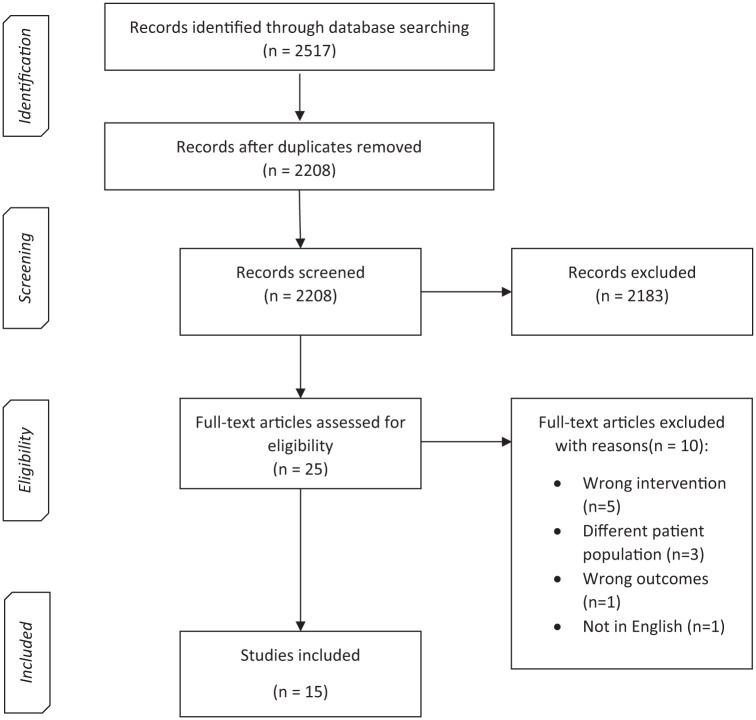
PRISMA flow diagram outlining study selection process. PRISMA, Preferred Reporting Items for Systematic Reviews and Meta-Analyses.

Our literature search identified 2517 unique citations, which were reduced to 2208 after removing duplicates (Figure 1). At the title and abstract screening stage, 2183 studies were excluded. The most common reasons for exclusion were: studies not specific to RCC (e.g., other solid tumors or hematologic malignancies), studies evaluating metastases to organs other than bone, studies without original clinical data (e.g., reviews, conference abstracts, editorials), and studies that did not report relevant outcomes of interest. The remaining 25 articles were assessed in full text. Of those, 10 studies were excluded due to: wrong intervention (*n* = 5), different patient population (*n* = 3), wrong outcomes (*n* = 1), not published in English (*n* = 1). A total of 15 studies met all inclusion criteria and were included for data extraction. A flow diagram describing the study selection process is shown in Figure 1.

### Study characteristics

We identified 15 studies of orthopedic interventions targeting bone metastatic disease in RCC patients,^34–48^ with a total of 1006 patients. The key features of these studies are presented in Table 1. The studies were characterized by their intervention type: nine studies utilized surgical methods only (i.e., orthopedic interventions) and six studies utilized both surgical and non-surgical methods (i.e., orthopedic ± targeted therapy, systemic therapy, or radiotherapy). While all studies reported survival outcomes, five studies reported reoperation and recurrence rates, and six studies described treatment complications. Twelve studies did breakdown into the number of bony metastatic sites (solitary or multiple BM) while the remaining did not. With respect to the study design, the 15 articles that were included in this study were predominantly retrospective. Forty-seven percent of the studies were conducted in Japan, with the remaining originating in Europe, Asia, and the USA. The study size ranged from 16 to 253 with an average of 90 patients. Studies that reported on patients with multiple metastases separately are represented by the total sites of metastasis. All studies had predominantly male population (75.4%). A full list of study characteristics is outlined below in Table 2.

**Table 1.** Key features of included studies (*n* = 15).

Study feature	Count (*n*)	Percentage
Study intervention type		
Surgical method only	9	60
Surgical and non-surgical methods	6	40
Study outcomes		
Survival outcomes	15	100
Recurrence/reoperation rates	5	33.3
Complications	6	40
Study population characteristics		
Reported number of bony metastasis sites	12	80
Did not report the number of bony metastasis sites	3	12
Publication year		
2021–2024	4	26.7
2017–2020	6	40.0
2014–2016	5	33.3

**Table 2.** Breakdown of individual study characteristics.

Author(s), year of publication	Origin	Methodology	Study size	Total sites of metastasis	Male:female ratio
Laitinen 2015^42^	UK, Finland, Sweden	Retrospective	*n* = 253	268	173:95
Kato 2016^41^	Japan	Retrospective	*n* = 36	36	26:10
Fukushima 2016^36^	Japan	Retrospective	*n* = 71	71	59:12
Du 2016^35^	Germany	Retrospective	*n* = 114	114	89:25
Higuchi 2018^39^	Japan	Retrospective	*n* = 30	30	25:05
Higuchi 2018^38^	Japan	Retrospective	*n* = 58	58	46:12
Dong 2020^34^	China	Retrospective	*n* = 168	168	141:27
Shankar 2020^46^	USA	Retrospective	*n* = 78	78	55:23
Kato 2021^40^	Japan	Retrospective	*n* = 65	65	53:12
Hara 2023^37^	Japan	Retrospective	*n* = 20	26	22:04
Scoccianti 2023^47^	Italy	Case Series	*n* = 16	16	12:04
Tsukamoto 2023^48^	Japan	Retrospective	*n* = 97	103	76:27
Ptashnikov 2020^45^	Russia	Retrospective	*n* = 100	100	76:24
Langerhuizen 2016^43^	USA	Retrospective	*n* = 183	183	127:56
Mosele 2017^44^	Italy	Retrospective	*n* = 63	63	39:24

With regards to study population (Table 3), most patients presented with multiple bone metastases (59.2%). Clear cell carcinoma was the most common subtype in all studies reporting histological subtypes (84.5%). Studies that did not include a histological subtype analysis are listed as NA. The most common site of non-skeletal metastasis was lung (38.5%). Other patterns of metastasis also included liver, brain, and lymph nodes. The use of bone remodeling therapy, such as denosumab, was reported in 20% of the studies. Studies that used adjuvant treatment but did not explicitly identify or mention the regimens are labelled as unspecified.

**Table 3.** Patient characteristics in included studies.

Author(s), year of publication	Number of bony metastasis sites	Histological subtype of renal cell carcinoma	Pattern of non-skeletal metastatic disease	Adjunctive bone remodeling therapy
Laitinen 2015^42^	Solitary (141), multiple (127)	NA	Lung, visceral	No
Kato 2016^41^	Solitary (13), multiple (11)	Clear cell (32), granular (1), papillary (1), chromophobe (1), sarcomatoid (1)	Lung, liver, lymph nodes, pancreas	No
Fukushima 2016^36^	Solitary (46), multiple (25)	NA	Not specified	Yes
Du 2016^35^	Solitary (68), multiple (46)	Clear cell (84), non-clear cell (21), not specified (9)	Lung, liver, lymph nodes, brain, adrenal gland	Yes
Higuchi 2018^39^	NA	Clear cell (28), Non-clear Cell (2)	Lung, visceral	Unspecified
Higuchi 2018^38^	NA	Clear cell (49), non-clear cell (5)	Lung	Unspecified
Dong 2020^34^	Solitary (50), multiple (118)	Clear cell (150), papillary (8), chromophobe (1), translocation RCC (1), not specified (8)	Lung, liver, brain, pancreas	Unspecified
Shankar 2020^46^	NA	Clear cell (72), granular (2), sarcomatoid (2), eosinophilic (1), chromophobe (1)	Lung, liver, brain, adrenal glands, pancreas	No
Kato 2021^40^	Solitary (24), multiple (19)	Clear cell (62), Non-clear cell (3)	Lung, liver, lymph nodes, pancreas, kidney	Unspecified
Hara 2023^37^	Solitary (8), multiple (18)	Clear cell (20)	Not specified	No
Scoccianti 2023^47^	Solitary (11), multiple (5)	Clear cell (16)	Lung	No
Tsukamoto 2023^48^	Solitary (43), multiple (54)	Clear cell (92) non-clear cell (5)	Not specified	Yes
Ptashnikov 2020^45^	Solitary (8), multiple (89)	NA	Lung, liver, lymph nodes, brain, kidneys	No
Langerhuizen 2016^43^	Solitary (44), multiple (97)	Clear cell (140), non-clear cell in (23)	Lung, liver, brain	No
Mosele 2017^44^	Solitary (4), multiple (59)	Clear cell (40), papillary (12), chromophobe (6), multilocular cystic (3), collecting duct (1), not specified (1)	Not specified	No

NA, not applicable.

### Results of individual studies

The following subsection has been organized according to the location of treated bone metastasis in each study. In Tables 4–6, surgical interventions are defined as orthopedic surgical approaches, whereas non-surgical interventions include targeted therapies, systemic therapies, and radiotherapy.

**Table 4.** Survival outcomes in bone metastasis to the appendicular skeleton.

Author(s), year of publication	Study group based on intervention type, for example, surgical, non-surgical, combinations	Primary outcome (overall survival)
Laitinen et al., 2015^42^	Surgical	MST (after operation): 14 months
Higuchi et al., 2018^39^	Surgical	MST: 4.4 years, with 3-, 5-, 10-, and 15-year OS rates of 76%, 48%, 35%, and 23%, respectively
Hara et al., 2023^37^	Surgical + targeted therapy	MST: 38 months, with 1-, 3-, and 5-year survival rates of 80.8, 51.2, and 39.5%, respectively
Scoccianti et al., 2023^47^	Surgical	OS: 60% and 35% at 3 and 5 years, respectively
Tsukamoto et al., 2023^48^	Surgical	Median OS: 15 months
Langerhuizen et al., 2016^43^	Whole study cohort	OS probability at 1 year: 62%
	Surgical: metastasectomy group	OS probability at 1 year: 57% *p* < 0.05
	Surgical: intralesional curettage group	OS probability at 1 year: 29% *p* < 0.05
	Surgical: stabilization only group	OS probability at 1 year: 23% *p* < 0.05

CSS, Cancer-specific survival; MST, Median survival time; OS, overall survival.

**Table 5.** Survival outcomes in studies of bone metastasis to axial skeleton.

Author(s), year of publication	Study group based on intervention type, for example, surgical, non-surgical, combinations	Primary outcome (survival)
Kato et al., 2016^41^	Surgical	Estimated median CSS: 130 months, with 3, 5, and 10-year CSS rates of 77.8%, 69.1%, and 58.0%, respectively
Shankar et al., 2020^46^	Whole study cohort	OS: 717 days
	Surgical only	OS: 222 days (*p* = 0.003)
	Surgical + postop targeted therapy	OS: 913 days (*p* = 0.003)
Kato et al., 2021^40^	Surgical	Estimated median CSS time: 100 months
Ptashnikov et al., 2020^45^	Surgical + targeted therapy	Median OS: 22 months, with 3- and 5-year survival rates of 29.7% and 12%, respectively
Mosele et al., 2017^44^	Surgical	MST: 26.9 months, with 1-, 2-, 3-, and 5-year OS rates of 49.20%, 31.74%, 12.26%, and 6.34%, respectively

CSS, Cancer-specific survival; MST, Median survival time; OS, overall survival.

**Table 6.** Survival outcomes in studies of bone metastasis to both appendicular and axial skeleton (mixed skeleton).

Author(s), year of publication	Study group based on intervention type, e.g., surgical, non-surgical, combinations	Primary outcome (survival)
Fukushima et al., 2016^36^	Whole study cohort	Median OS: 25 months
	Surgical: en bloc resection	OS for patients with solitary lesions: 27 months
	Surgical: curettage	OS for patients with solitary lesions not reached
	Non-surgical: radiotherapy (RT) with BED > 85 Gy	OS for patients with solitary lesions: 53 months
	Non-surgical: radiotherapy (RT) with BED < 85 Gy	OS for patients with solitary lesions: 10 months
Higuchi et al., 2018^38^	Whole study cohort	MST of all patients was 100 months, with 1-, 3-, 5-, 10-, and 15-year OS rates of 89%, 75%, 62%, 48%, and 25%, respectively
	Surgical: en bloc metastasectomy	MST: 127 months (*p* = 0.29)
	Surgical: intralesional curettage	MST: 54 months (*p* = 0.29)
Dong et al., 2020^34^	Whole study cohort	Median OS: 43 months, with 1-, 3-, and 5-year OS rates of 77.4%, 55.9% and 31.8%, respectively
	Surgical + adjuvant targeted therapy	Median OS: 54.7 months (*p* = 0.787)
	Surgical without adjuvant targeted therapy	Median OS: 51.3 months (*p* = 0.787)
Du et al., 2016^35^	Whole study cohort	Median OS was 9.6 months
	Resection of bone metastasis with targeted therapy (resection before targeted therapy)	Median OS: 31.8 months
	Targeted therapy (TT) only with no resection	Median OS: 7.6 months
	Radiotherapy (RT) groups: RT only, RT + TT, RT + BM resection, RT + BM resection + TT	There was no difference of OS between patients with and without RT

BM, bone metastasis; CSS, Cancer-specific survival; MST, Median survival time; OS, overall survival.

#### Survival outcomes in appendicular skeleton

For the appendicular skeleton (Table 4), survival outcomes varied across studies and intervention types. Median OS ranged from 14 months to 4.4 years for surgical-only interventions. Laitinen et al.^42^ reported a median survival time (MST) of 14 months post-surgery, while Higuchi et al. documented a much longer MST of 4.4 years, with long-term OS rates of 76%, 48%, and 35% at 3, 5, and 10 years, respectively.^39^ Hara et al.^37^ noted improved outcomes with a combination of surgical and targeted therapy, showing an MST of 38 months and 1-, 3-, and 5-year OS rates of 80.8%, 51.2%, and 39.5%, respectively. Langerhuizen et al.^43^ highlighted significant differences between surgical approaches, where metastasectomy resulted in superior 1-year OS (57%) compared to intralesional curettage (29%) and stabilization-only (23%), with *p* < 0.05.

#### Survival outcomes in axial skeleton

With respect to the axial skeleton (Table 5), survival metrics included both OS and cancer-specific survival (CSS). Surgical-only interventions yielded variable MSTs, ranging from 222 days^46^ to over 100 months.^40,41^ Kato et al. demonstrated high CSS values, with a median CSS of 130 months in one study and 100 months in another, alongside long-term survival rates of 77.8%, 69.1%, and 58% at 3, 5, and 10 years, respectively.^40,41^

Combined therapies were associated with significant survival advantages. For instance, Shankar et al.^46^ reported an MST improvement from 222 days for surgery alone to 913 days when postoperative targeted therapy was included (*p* = 0.003). Ptashnikov et al.^45^ observed a median OS of 22 months with combined surgical and targeted therapy, while Mosele et al.^44^ reported an MST of 26.9 months for surgical interventions, with 5-year survival rates as low as 6.34%.

#### Survival outcomes in mixed skeleton

Within mixed skeleton studies (Table 6), survival outcomes varied significantly based on the intervention type. Fukushima et al. showed MSTs ranging from 10 months with low-dose radiotherapy (biologically effective dose [BED] < 85 Gy) to 53 months with high-dose radiotherapy (BED > 85 Gy).^36^ In terms of surgical approaches, Higuchi et al.^38^ reported an MST of 127 months for en bloc metastasectomy compared to 54 months for intralesional curettage (*p* = 0.29, not statistically significant). When surgery and systemic therapy were utilized, there were some interesting findings. For instance, Dong et al.^34^ observed MSTs of 54.7 months for surgery with targeted therapy versus 51.3 months for surgery alone (*p* = 0.787, not statistically significant). Du et al.^35^ noted that combining surgery with targeted therapy achieved an MST of 31.8 months, while targeted therapy alone resulted in 7.6 months.

#### Surgical versus combined therapies and non-surgical interventions

In axial skeleton cases, Shankar et al.^46^ showed a significant increase in MST from 222 days with surgery alone to 913 days with added targeted therapy (*p* = 0.003). Similarly, Du et al.^35^ highlighted a significant OS improvement (31.8 months) with surgery and targeted therapy compared to targeted therapy alone (7.6 months), although *p*-values were not reported. Non-surgical interventions like high-dose radiotherapy also achieved competitive results, particularly for isolated metastases in mixed skeleton cases (MST of 53 months, BED > 85 Gy).^36^

#### Differences among surgical interventions

For appendicular skeleton metastases, Langerhuizen et al.^43^ demonstrated significantly higher 1-year OS probabilities for complete metastasectomy (57%) compared to intralesional curettage (29%) and stabilization-only surgery (23%) (*p* < 0.05). In mixed skeleton cases, Higuchi et al.^38^ reported an MST of 127 months for en bloc metastasectomy versus 54 months for intralesional curettage, though the difference was not statistically significant (*p* = 0.29).

## Discussion

### Survival outcomes based on intervention type

The findings of this systematic review highlight the fact that orthopedic interventions remain central in the management of bone metastases in patients with renal cell carcinoma. Our analysis suggests that surgical intervention generally outperforms non-surgical treatments or adjuvant therapy alone in terms of survival outcomes, particularly when combined with systemic therapy. This benefit was particularly evident in patients who received targeted therapy following metastasectomy, underscoring the value of combining surgical and targeted therapies for optimal outcomes.^46^ Complete resection of metastasis has been shown to offer survival benefits for RCC metastasis to any organ.^41^ In several studies, complete metastasectomy was associated with longer survival than stabilization or intralesional curettage.^36–38,41^ However, it is important to note that all included studies were retrospective or case series, with small cohorts, limited statistical rigor, and strong potential for selection bias. Thus, while a survival signal exists, causality cannot be established.

Immune checkpoint inhibitor (IO)–based regimens, including IO–IO and IO–TKI combinations, now define first-line therapy for metastatic RCC. Subgroup analyses from CheckMate 214^9^ and CheckMate 9ER^12^ confirm that outcomes for patients with bone metastases remain inferior despite systemic advances, underscoring the unmet need in this subgroup. These findings reinforce that bone metastases remain an adverse prognostic factor and highlight the need to contextualize surgical decisions within systemic disease management. Combined approaches (surgery + systemic therapy) may be reasonable in highly selected patients, but robust evidence is lacking.

In terms of non-surgical strategies, one study showed that high-dose radiotherapy demonstrated promising results in comparison to low-dose radiotherapy, highlighting the dose-response relationship.^36^ While radiotherapy can serve as a viable alternative for patients unfit for surgery, the outcomes generally favor surgical, or combined approaches for a better and durable disease control. Patients who may require careful evaluation for local surgery include patients in the Memorial Sloan–Kettering Cancer Center and International Metastatic RCC Database Consortium poor risk categories, who demonstrated worse prognoses than intermediate-risk groups in Dong et al.’s^34^ study.

### Solitary versus multiple and synchronous versus metachronous bone metastases

Across several studies, solitary bone metastasis was consistently associated with longer survival after surgery compared with multiple lesions. For instance, Laitinen et al.^42^ and Mosele et al.^44^ both found that patients with solitary bone metastasis who underwent surgery demonstrated significantly increased overall survival relative to patients with multiple metastases in their study populations. This finding was also supported by Dong et al.^34^ on multivariable analysis, though the number of bone metastases did not lead to significance with overall survival on univariate analysis. Fukushima et al.^36^ showed that patients treated with intensive local therapy (defined as en bloc resection, curettage, or RT with BED > 85 Gy) demonstrated significantly better OS (*p* = 0.006) for those with solitary bone metastasis, but only marginal significance (*p* = 0.052) for those with multiple bone metastases, when compared to those treated with less intensive (RT with BED < 85 Gy) or no therapy. Reserving metastasectomy to those with solitary resectable lesions is consistent with the National Comprehensive Cancer Network (NCCN) guidelines for kidney cancer.^49^ Nevertheless, Higuchi et al.^39^ assert that metastasectomy of multiple metastases can still be a reasonable option that provides relatively long survival, albeit less than solitary lesions. For instance, in the study, while mean survival was significantly lower in patients who underwent metastasectomy with more than two metastatic sites as compared to one metastatic site, it was still favorable to that reported in past literature.^39^

An additional nuance is the distinction between synchronous and metachronous bone metastases. Several series suggested that metachronous lesions are associated with longer survival, potentially reflecting less aggressive tumor biology.^35,42,44^ Unfortunately, not all included studies reported this distinction, which represents a limitation of the current evidence.

### Bone-modifying therapy use

Bone-modifying therapy (BMT), such as bisphosphonate or denosumab, has been established as an important player in reducing skeletal-related events across malignancies. Yet, they were under-reported or under-utilized in the majority of included studies. Eight of the fifteen studies in this systematic review did not mention any use of BMT alongside the orthopedic interventions. Four studies mentioned the use of adjunctive therapies but did not specify which regiments or their effects on the study outcomes.^34,36–38^ Bone-modifying therapy was only explicitly mentioned in three of the studies, and included treatments such as denosumab and zoledronic acid, among others.^35,36,48^

In patients with recurring skeletal metastases following orthopedic interventions, bisphosphonates were given to prevent further local regression.^35,36,48^ The findings were inconclusive as this was done to control local regression and improve patient quality of life, rather than explicitly investigate the effects of bisphosphonate use on local recurrence.^35,36,48^ Overall, the studies did not demonstrate survival improvement with the administration of bone-modifying therapy,^35,36,48^ but this may be due to limited reporting, heterogeneity, and lack of dedicated analysis. Future studies should systematically evaluate BMT use in RCC bone metastases, ideally in the context of modern systemic regimens.

### Complications, recurrence, and reoperation rates

With regards to unwanted outcomes from the studied interventions, 9 of the 15 included studies discussed one or more of complications, recurrence (of metastasis), and/or reoperation rates. The incidence of complications ranged from 11.1% to 38%,^35,38–40,42,43^ including fractures, infections, nerve palsies, vessel injury, and infarctions. These data do not differentiate well the rates of the complications across various interventions (surgical vs chemotherapy or radiation, or even between surgical interventions). Only one study, presented by Higuchi et al.,^39^ conducted a multivariate analysis in this area and suggested that only intralesional resection was an independent risk factor for poor prognosis. Additionally, limited literature in the context of interventions on bone metastasis of RCC makes it difficult to gauge the clinical significance of these findings (i.e., whether these are higher or lower compared to other interventions).

Only 5 of the 15 studies included reported outcomes related to local recurrence or tumor progression requiring reoperation, with incidences ranging from 0% to 19%.^35,40,41,43,46^ Langerhuizen et al.^43^ further distinguished between local tumor recurrence and reoperation rate, suggesting that recurrence/local tumor progression is significantly lower after metastasectomy, followed by intralesional curettage, and then stabilization interventions alone. However, this trend did not translate into differences in reoperation rates, which were similar across surgical techniques. This discrepancy highlights the complexity of interpreting recurrence versus surgical revision, as reoperation may be influenced not only by local tumor control but also by patient fitness, expected prognosis, and multidisciplinary decision-making.

### Quality of life

Assessment of quality of life (QoL) outcomes in patients with RCC and bone metastasis was limited across the included studies. Despite this paucity of data, the evidence available suggests a potential positive impact of orthopedic interventions on functional recovery and symptom relief.

Shankar et al.^46^ reported that 93% of patients presenting with myelopathy or weakness secondary to epidural disease experienced postoperative improvement in ambulation and strength. Similarly, Scoccianti et al.^47^ observed complete gait recovery in all patients following surgery. These findings, although derived from small retrospective cohorts, highlight the role of orthopedic surgery in restoring mobility and preserving independence in a population at high risk of functional decline.

Nevertheless, the absence of standardized QoL assessments—such as validated patient-reported outcome measures—limits the strength of these conclusions. Further prospective studies integrating formal QoL endpoints are required to better quantify the benefits of surgery beyond survival and local control.

### Limitations of systematic review

Several limitations can be addressed in this systematic review. Firstly, most of the studies were retrospective in nature, which limits their generalizability and impact. The lack of randomized control trial limits the reproducibility and statistical significance of these findings. A dedicated randomized control trial would be beneficial to draw solid conclusions with minimal bias and confounding variables. Furthermore, the countries of origin were generally of a higher socioeconomic status, which further limits the generalizability of the findings on a global scale. The studies also had inconsistent sample sizes, which makes it difficult to compare the results accurately. Some studies had sample sizes that were too small to draw any solid conclusions. Only a minority of patients in the included studies received bone-targeted therapies such as bisphosphonates or denosumab. This underutilization represents a significant limitation, as these agents are known to reduce skeletal-related events and may influence outcomes. Moreover, the inconsistency between studies in their use of bone-modifying therapy introduces a major confounding factor. In studies where bone-modifying therapies were used, administration was not randomized, but limited to patients with recurrent local metastasis for QoL maintenance.

### Future directions

The current evidence on orthopedic interventions for patients with RCC with metastasis to the bones is limited, consisting almost exclusively of retrospective studies and case series with mostly narrative results. As such, conclusions regarding a survival benefit from surgery remain speculative and prone to selection bias.

Future research should aim to address these limitations through prospective, ideally RCTs, or at least large multicenter retrospective studies with the use of more inferential and robust statistical methods. Such studies should focus on clinically meaningful and consistent outcomes, including overall survival, complications, reoperation rates, and measures of quality of life or functioning.

Given the systemic nature of RCC, even in cases of oligometastatic disease to the bones, further analyses must also integrate the role of modern systemic therapy. Comparative studies examining orthopedic surgery versus radiation, as well as head-to-head evaluations of different surgical approaches, would be valuable to clarify their respective roles.

Ultimately, orthopedic interventions should be studied not in isolation but as part of a multidisciplinary care model that includes systemic therapy, radiotherapy, and supportive measures. Building the literature in this direction will help establish evidence-based recommendations, with the goal of improving both survival and quality of life for patients with RCC and bone metastases.

## Conclusion

Our systematic review highlights the lack of high-quality evidence regarding the role of orthopedic surgery in managing RCC bone metastases. All available studies were retrospective or small case series, with outcomes primarily reported narratively and limited statistical analysis. While some reports suggest an association between complete metastasectomy and longer survival, these findings are subject to strong selection bias and cannot establish causality.

Bone metastases remain a well-known adverse prognostic factor in RCC, and even in cases of solitary bone involvement, the disease should be considered systemic. Importantly, the treatment paradigm for metastatic RCC has shifted with immunotherapy-based combinations, and subgroup analyses from contemporary randomized trials demonstrate that patients with bone metastases continue to experience inferior outcomes compared with those without bone disease.

Orthopedic interventions may provide meaningful symptomatic relief, structural stability, and improved function and should be considered as part of a multidisciplinary, individualized treatment strategy. However, their role in prolonging survival remains unproven. Given the heterogeneity of study methodologies and the predominance of retrospective data, further research—particularly randomized controlled trials—will be essential to optimize patient selection, clarify the value of orthopedic surgical intervention relative to other local therapies such as radiotherapy, and establish evidence-based guidelines for the management of RCC patients with bone metastases.

## Declarations

**Ethics approval and consent to participate:** Not applicable.

**Consent for publication:** Not applicable.

**Availability of data and materials:** All data generated or analyzed in this study are included in this published article. This review was not registered.
